# HPMC/PVP K90 Dissolving Microneedles Fabricated from 3D-Printed Master Molds: Impact on Microneedle Morphology, Mechanical Strength, and Topical Dissolving Property

**DOI:** 10.3390/polym16040452

**Published:** 2024-02-06

**Authors:** Baramee Chanabodeechalermrung, Tanpong Chaiwarit, Siripat Chaichit, Suruk Udomsom, Phornsawat Baipaywad, Patnarin Worajittiphon, Pensak Jantrawut

**Affiliations:** 1 Department of Pharmaceutical Sciences, Faculty of Pharmacy, Chiang Mai University, Chiang Mai 50200, Thailand; barameechana@gmail.com (B.C.); tanpong_c@cmu.ac.th (T.C.); siripat.chaichit@cmu.ac.th (S.C.); 2 Biomedical Engineering and Innovation Research Center, Chiang Mai University, Chiang Mai 50200, Thailand; suruk_u@cmu.ac.th (S.U.); phornsawat.b@cmu.ac.th (P.B.); 3 Biomedical Engineering Institute (BMEI), Chiang Mai University, Chiang Mai 50200, Thailand; 4 Office of Research Administration, Chiang Mai University, Chiang Mai 50200, Thailand; 5 Department of Chemistry, Faculty of Science, Chiang Mai University, Chiang Mai 50200, Thailand; patnarin.w@cmu.ac.th; 6 Center of Excellence in Materials Science and Technology, Chiang Mai University, Chiang Mai 50200, Thailand; 7 Center of Excellence in Agro Bio-Circular-Green Industry (Agro BCG), Agro-Industry, Chiang Mai University, Chiang Mai 50100, Thailand

**Keywords:** dissolving microneedles, 3D printing, lidocaine HCl, intradermal drug delivery, hydroxypropyl methylcellulose, PVP K90

## Abstract

Three-dimensional (3D) printing can be used to fabricate custom microneedle (MN) patches instead of the conventional method. In this work, 3D-printed MN patches were utilized to fabricate a MN mold, and the mold was used to prepare dissolving MNs for topical lidocaine HCl (L) delivery through the skin. Topical creams usually take 1–2 h to induce an anesthetic effect, so the delivery of lidocaine HCl from dissolving MNs can allow for a therapeutic effect to be reached faster than with a topical cream. The dissolving-MN-patch-incorporated lidocaine HCl was constructed from hydroxypropyl methylcellulose (HPMC; H) and polyvinyl pyrrolidone (PVP K90; P) using centrifugation. Additionally, the morphology, mechanical property, skin insertion, dissolving behavior, drug-loading content, drug release of MNs and the chemical interactions among the compositions were also examined. H5_1_P_2_-L, H50_1_P_2_-L, and H90_1_P_2_-L showed an acceptable needle appearance without bent tips or a broken structure, and they had a low % height change (<10%), including a high blue-dot percentage on the skin (>80%). These three formulations exhibited a drug-loading content approaching 100%. Importantly, the composition-dependent dissolving abilities of MNs were revealed. Containing the lowest amount of HPMC in its formulation, H90_1_P_2_-L showed the fastest dissolving ability, which was related to the high amount of lidocaine HCl released through the skin. Moreover, the results of an FTIR analysis showed no chemical interactions among the two polymers and lidocaine HCl. As a result, HPMC/PVP K90 dissolving microneedles can be used to deliver lidocaine HCl through the skin, resulting in a faster onset of anesthetic action.

## 1. Introduction

A microneedle (MN) patch is a small device comprising various microscale needles, generally ranging in height from 25 to 2000 μm and with different shapes, which individually arrange on a base support [1]. Utilizing MNs can prevent the contact of nerve fibers and blood vessels located in the dermis layer, reducing a patient’s pain, so MNs have been developed to solve the problem of pain concerns, increasing patient compliance, which can also improve the treatment efficacy [2,3]. Moreover, the delivery of drug using MNs can avoid first-pass metabolism in the liver, resulting in escalating bioavailability [4]. Ideally, MNs piercing through the skin create various micro-holes, allowing drugs or large substances to be delivered through these micro-holes [5]. Typically, MNs are manufactured from different materials, such as metals, polymers [2], and silica [1,6]. Depending on the delivery approaches, MNs can be divided into five categories: solid MNs, hollow MNs, coated MNs, dissolving MNs, and hydrogel-forming MNs [2,7].

Dissolving MNs are produced from water-soluble materials, such as hyaluronic acid, maltose, polyvinyl pyrrolidone (PVP) [5], sucrose [3], and hydroxypropyl methylcellulose (HPMC) [8], and these five polymers have no toxicity and are safe to use in humans [4,6,9]. The drugs incorporated in dissolving MNs are dissolved or dispersed in the needles [5]. After skin insertion, the dissolving MNs should be able to dissolve after contacting water or biological fluid [5,8] and release the drugs through the skin with none of the original shape of the MN left behind [9]. Regarding their versatility, dissolving MNs have been developed to deliver various drugs, such as propranolol hydrochloride [5] and donepezil hydrochloride [8], as well as macromolecules of DNA, RNA, and proteins [6,10]. The conventional method utilized to prepare MN patches is through micro-electromechanical systems (MEMS) or micro-machining; although this is a potential tool for mass production, this technique requires highly specialized training and has complex multi-step processes for production, unlike the three-dimensional (3D) printing technique [1].

Three-dimensional printing is a modern technique with layer-by-layer object manufacturing [11]. A 3D object is initially designed using computer-aided design (CAD) software. The designed scheme is then sliced into layers. Each layer is subsequently printed by a 3D printer connected to the computer for 3D scheme designing [6,11,12]. Three-dimensional printing technology contributes to object fabrication with a high complexity in a fast and cost-effective way. Interestingly, the use of vat photopolymerization techniques, such as stereolithography (SLA) and digital light processing (DLP), has been reported for constructing MNs [10]. Han et al. directly constructed backward-facing barbs on MNs using a DLP technique to increase skin adhesion, and the results showed that the barbs improved skin adhesion and could be used for sustained drug release. However, the use of Sudan I as a photoabsorber in the polymer resin renders it toxic to humans [13], since Sudan I is recognized as a harmful substance that leads to DNA damage, resulting in genotoxic effects. Moreover, Sudan I has been frequently reported as a carcinogen [14,15]. Additionally, utilizing an SLA technique instead of a DLP technique reduces the cost of production, as SLA printers are cheaper than DLP printers, and the resolution of the 3D-printed objects is not different, whereas the printing process of DLP printers is faster than that of SLA printers [16]. In addition, 3D printing can be used to construct MN patches, and the MN molds are fabricated from those of 3D-printed MN patches; thus, the concern regarding polymer resin toxicity is eradicated [17]. Therefore, the use of 3D printing shows benefits over the conventional method, MEMS, including a fast production speed, the ability to easily scale up, and no required special training [1].

Lidocaine, a common anesthetic agent, is widely used to numb a small area of the body temporarily before providing a tattoo, performing plastic surgery, or extracting teeth in a dental operation [18]. Lidocaine-based drugs are insoluble in water. The hydrochloride (HCl) salt form (lidocaine HCl) is generally used worldwide due to its excellent water solubility. Moreover, lidocaine HCl shows a superior safety profile over other anesthetic agents; thus, it has been applied in clinical practice and can be used in acute and chronic pain as an adjuvant analgesic agent [19]. However, lidocaine HCl is categorized in the biopharmaceutics classification system (BCS) as class III, and it has a high solubility but a low permeability. Lidocaine HCl is generally administered via an intravenous or intramuscular injection to provide a high bioavailability, but it causes pain during insertion [18]. Due to its poor permeability, the slow penetration of lidocaine HCl is a major drawback of its topical use, since it typically takes at least ~1–2 h to reach a therapeutic effect [20]. However, lidocaine HCl shows a rapid onset of action (~20–60 s) [21], which is promising for its use with dissolving MNs, which can rapidly provide the therapeutic anesthetic effect of lidocaine HCl through the skin without pain during skin insertion.

In this study, 3D printing was used to print an MN master, which is followed by a construction of polydimethylsiloxane (PDMS) reverse mold using the 3D-printed MN master as a template. After that, solutions of HPMC, PVP K90, and their blends were used as polymeric materials to prepare dissolving MN patches. The formulations exhibiting proper physicochemical properties were then selected for lidocaine HCl incorporation to evaluate their drug loading and dissolving capacity, including drug release behavior and components’ chemical interactions.

## 2. Materials and Methods

### 2.1. Materials

Acrylic-based resin (eResin PLA biophotopolymer resin) was purchased from eSun Industrial Co., Ltd., Shenzhen, China). Polydimethylsiloxane (PDMS) was purchased from Dow Deutschland Inc., Berlin, Germany. Hydroxypropyl methylcellulose (HPMC) E5 and HPMC E50 were purchased from Onimax Co., Ltd., Bangkok, Thailand. HPMC 90SH were purchased from Shin-Etsu Chemical Co., Ltd., Tokyo, Japan. Polyvinylpyrrolidone (PVP) K90 and isopropanol were purchased from Union Science Co., Ltd., Chiang Mai, Thailand. Ethanol was purchased from RCI Labscan, Ltd., Bangkok, Thailand. Lidocaine HCl was purchased from S. Tong chemical Co., Ltd., Nonthaburi, Thailand. Deionized water (DI) served as a solvent to prepare microneedle patches.

### 2.2. 3D Printing of Microneedle Masters

**Figure 1:**
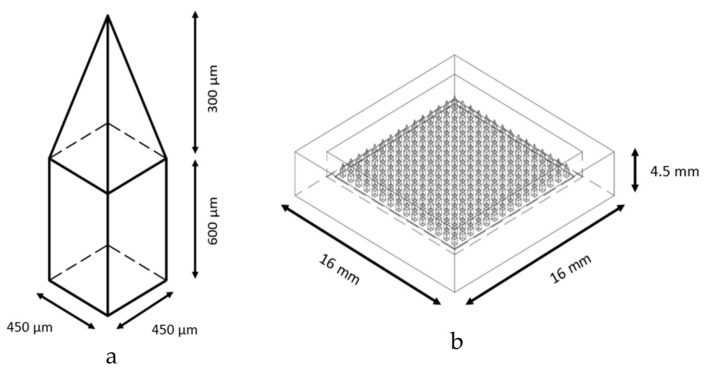
The design of microneedle (**a**) and microneedle patch (**b**) using Fusion 360 software version 2.0.16985.

Designed microneedle patches were printed using an LCD-based SLA 3D Printer (ANYCUBIC Photon, Anycubic Technology, Hong Kong, China). Briefly, CAD files of MN patches were produced using Fusion 360 software version 2.0.16985 (Autodesk Incorporation, San Rafael, CA, USA). MN was designed into a square pyramidal shape with 900 µm height and 450 µm width at the base (Figure 1a). Each MN patch consisted of an array of 15 × 15 needles with 500 µm spacing between the needles on a 16 × 16 × 2 mm base (Figure 1b). MN patches were fabricated from acrylic-based resin (eResin PLA biophotopolymer resin, eSun Industrial Co., Ltd., Shenzhen, China). After fabrication, the MN patches were shortly rinsed with isopropanol to remove residual resin and post-cured for 10 min using an ANYCUBIC Cure Machine 2.0 ultraviolet light emitting diode (LED) lamp (Anycubic Technology, Hong Kong, China).

### 2.3. Fabrication of Reverse PDMS Microneedle Molds

To fabricate a reverse polydimethylsiloxane (PDMS) MN mold, liquid phase PDMS (elastomer: curing agent = 10:1 *w*/*w*) was first cast onto a 3D-printed MN master (15 × 15 pyramidal tip array, 450 μm base width, 900 μm height). After curation at 60 °C for at least 4 h, an inverse PDMS replica was fabricated after carefully tearing off from the MN master. After obtaining an MN master mold, the mold was cut for measurements of the height and width of the needle hole using a scanning electron microscope (SEM) (JEOL JCM-7000 NeoScopeTM Benchtop, Tokyo, Japan) operated at 15 kV under low vacuum mode at 30× magnification.

### 2.4. Preparation of HPMC-Based Solutions

HPMC E5, HPMC E50, and HPMC 90SH solutions were separately dissolved in ethanol and DI water mixed in a ratio of 8:2 at the concentration of 20, 7, and 1.5% *w*/*w*, respectively, using magnetic stirring. To prepare HPMC/PVP K90 solutions, each HPMC E5, HPMC E50, and HPMC 90SH solution was individually mixed with 40% *w*/*w* PVP K90 solution. Then, the PVP K90 was previously dissolved in the solvent mentioned above at the weight ratios of 1:1, 1:2, and 2:1. Moreover, the amount of raw material used in each formulation is shown in Table 1.

### 2.5. Fabrication of HPMC/PVP K90 Microneedle Patch

To prepare an HPMC/PVP K90 MN patch, an MN mold and a polymer solution were put into a centrifuge tube, and the solution was forced into the mold cavity by a centrifuge machine (MPW-352R, Warsaw, Poland) at 6000 rpm for 2 h. It is noted that all aqueous solutions were previously centrifuged at 6000 rpm for 10 min to completely remove air bubbles. After 2 h, the MN mold was taken out, which was followed by removal of the excess polymer solution. The polymer-packed mold was then dried to form an MN patch at room temperature for 24 h. Finally, the MN patch was peeled off from the PDMS mold and stored in a dry ambient condition before use.

### 2.6. Physicochemical Properties of Microneedle Patch

#### 2.6.1. Rheological Property of the Polymer Solutions

The polymer solutions were subjected to rheological behavior investigation using a Brookfield Rheometer (R/S-CPS, P25 DIN plate, Brookfield Engineering Laboratories, Middleboro, MA, USA) via parallel plates with a diameter of 25 mm and a 1 mm plate gap. In each measurement, a sample of ~0.6 mL was used to evaluate the rheological behavior.

#### 2.6.2. Morphological Structure Investigation

The MN structure was evaluated using a scanning electron microscope (JEOL JCM-7000 NeoScopeTM Benchtop, Tokyo, Japan) at 15 kV under low vacuum mode. The prepared MN patch was cut and mounted on an aluminum stub with double-sided adhesive carbon tape. Gold-sputtered coating was performed for all MN samples for 1 min prior to the SEM operation at 30× magnification.

#### 2.6.3. Mechanical Property Study

The mechanical strength of MN was evaluated by a Texture Analyzer, TA.XTplusC (Stable Micro Systems, Surrey, UK) in a compression mode. Firstly, a photograph of MN was taken for a measurement of the MN height (H_1_) before the compression using ImageJ software version 1.80. The MN patch was then attached to the movable probe using double-sided adhesive tape. Subsequently, the probe was compressed to a stainless-steel platform at a speed 0.5 mm/s until reaching the maximum force, which was from 0.028 to 0.11 N/needle. Moreover, the pre-test and post-test speed were predetermined at 1 mm/s, and the trigger force was 0.049 N. Finally, another photograph of the MN was taken to determine the height after compression (H_2_). The % height change in the MN was calculated using Equation (1).

(1) $$\%\;\mathrm{Height}\;\mathrm{change}=\frac{H_{1}-H_{2}}{H_{1}}\times 100$$

#### 2.6.4. Ex Vivo Skin Insertion Study

To evaluate the skin penetration test, neonatal porcine skin was used to substitute human skin. Moreover, the natural death piglets were fresh from a local farm in Lamphun, Thailand. Firstly, neonatal porcine skin was washed with phosphate-buffered saline (PBS) (pH 7.4) and then gently blotted with filter paper to dry the skin. Secondly, MNs were inserted into the skin using a thumb press on the MN patch for 60 s. Then, 0.1% *w*/*v* methylene blue was applied on the skin. After 10 min, PBS pH 7.4 was utilized to wash methylene blue. Lastly, the blue-dot percentage (% blue dots) that appeared on the skin was calculated using the following Equation (2).

(2) $$\%\;\mathrm{Blue}\;\mathrm{dots}=\frac{The\;number\;of\;blue\;dots\;appeared\;on\;the\;skin}{The\;number\;of\;needles\;of\;MN}\times 100$$

### 2.7. Preparation of Lidocaine HCl-Incorporated Microneedle Patch

After physicochemical property examination, the formulations showing the proper physicochemical properties (completed shape of needle, sharp tips, low % height change, and high % number of blue dots) were selected for the incorporation of 5% *w*/*w* lidocaine HCl and -L after those of the selected formulation referred to lidocaine HCl. Lidocaine HCl was added to the polymer solution with a magnetic stirrer until the solution was homogeneous. After that, the polymer solution was forced into the mold cavity using the method mentioned in Section 2.5.

### 2.8. Characterization of Lidocaine HCl-Loaded Microneedle Patch

After lidocaine HCl-incorporated MN patches were prepared, the MN patches were subjected to physical appearance investigation, a mechanical property test, and ex vivo skin insertion study using the method described in Section 2.6.2, Section 2.6.3 and Section 2.6.4 to ensure that MN patches possess the same properties as the blank MN patches.

### 2.9. Drug-Loading Content

Three random MN patches for each formulation were dissolved in DI water (10 mL) in a beaker under magnetic stirring. After that, the average amount of lidocaine HCl was examined using a UV-spectrophotometer (UV 2600i, Shimadzu Corporation, Kyoto, Japan) at 263 nm. The contents of lidocaine HCl were calculated from the standard curve of lidocaine HCl (0.125–0.625 μg/mL) with a high linear regression (r^2^ = 0.998) according to Equation (3).

(3) $$\%\;\mathrm{Drug}\;\mathrm{loading}\;\mathrm{content}=\frac{The\;amount\;of\;lidocaine\;HCl\;in\;MN\;patch}{Theoretical\;amount\;of\;lidocaine\;HCl\;in\;dry\;polymer}\times 100$$

### 2.10. Dissolving Study of Microneedle Patch

To evaluate their dissolving ability, MN patches incorporated with lidocaine HCl were investigated. Neonatal skin without a subcutaneous layer was used to study the MNs. Neonatal skin was applied on tissue paper saturated with PBS (pH 7.4) at 37 °C, and then the MN patches were applied on neonatal skin. After 5, 15, 30, and 60 min, the MN patches were removed for taking photos and measurements of the MN height using an RS PRO USB digital microscope (RS PRO, Bangkok, Thailand).

### 2.11. Lidocaine HCl Release

Lidocaine HCl release was performed using neonatal porcine skin as a membrane and PBS pH 7.4 as a biological fluid following the previously modified method [22]. The MN patch was pierced through neonatal porcine skin in the middle of the skin. After 1 h, the MN patch was taken out of the skin, and the piercing area was cut into a round shape with the diameter of 1.5 cm, and the area was 1.77 cm^2^. After that, the skin was cut into small pieces and then put into a 15 mL centrifuge tube with 5 mL of PBS pH 7.4. Then, it was sonicated using a sonicator (Elmasonic S100H, Elma, Singen, Germany) for 15 min followed by centrifuge using a centrifuge machine (MPW-352R, Warsaw, Poland) at 2000 rpm for 10 min. Finally, a UV-spectrophotometer (UV 2600i, Shimadzu Corporation, Kyoto, Japan) was utilized to evaluate the amount of released lidocaine HCl in the skin at 263 nm.

### 2.12. Fourier Transform Infrared Spectroscopy

Chemical interactions among the raw materials used in the formulations were determined using a Fourier transform infrared (FTIR) spectrometer (FT/IR-4700, Jasco, Tokyo, Japan). All formulations and raw materials were scanned in transmittance mode from 400 to 4000 cm^−1^ at a resolution of 4 cm^−1^.

### 2.13. Statistical Analysis

The results were reported as mean ± standard deviations (S.D.) using a one-way ANOVA test to evaluate the significant difference among the data via SPSS software version 17.0 (IBM Corporation, Armonk, NY, USA). The results showed a statistically significant difference when the *p*-value is less than 0.05.

## 3. Results and Discussion

### 3.1. 3D Printing of MN Master

**Figure 2:**
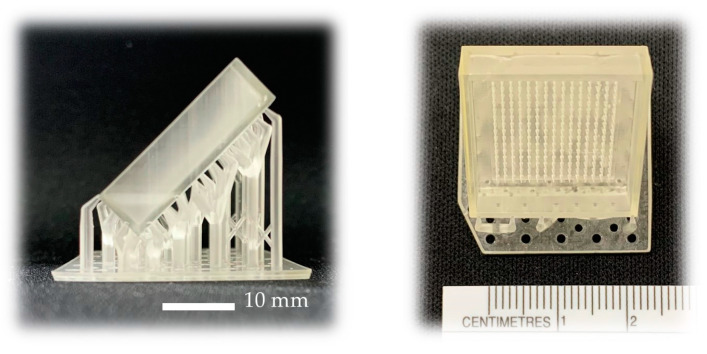
The 3D-printed MN patch master using an LCD-based SLA 3D Printer.

The MN patch master was printed using an LCD-based SLA 3D Printer (ANYCUBIC Photon, Anycubic Technology, Hong Kong, China). Each 3D-printed MN patch master consisted of an array of 15 × 15 needles with no structural break (Figure 2), which was similar to the design from Fusion 360 software. Moreover, MN patches were printed with the angle of 45° to make the tips sharper, since the layers of printing at 45° are more than 0°, resulting in the high accuracy of the 3D-printed object [23].

### 3.2. Fabrication of Reverse PDMS MN Mold

**Figure 3:**
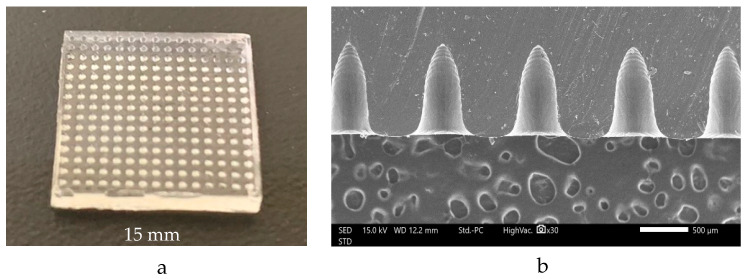
MN mold (**a**) and MN cavities in the mold (**b**) using SEM at 30× magnification.

Liquid-phase PDMS mixed with a curing agent at the ratio of 10:1 was cast on the MN patch and incubated in the oven at 60 °C to fabricate the MN mold. The results showed that the PDMS mold was clear and transparent with 15 × 15 needles cavities, as shown in Figure 3a. Moreover, the SEM image of the MN mold cavities (Figure 3b) showed the height and the width of the MN cavities, and the width of the needles was measured at half of the height. The results illustrated that the average height and width of the needles were 897.76 ± 21.04 and 449.64 ± 15.66 μm, respectively, which were approximately the same as the design (450 μm of width and 900 μm of height).

### 3.3. Physicochemical Properties of Microneedle

#### 3.3.1. Rheological Property of the Polymer Solutions

**Figure 4:**
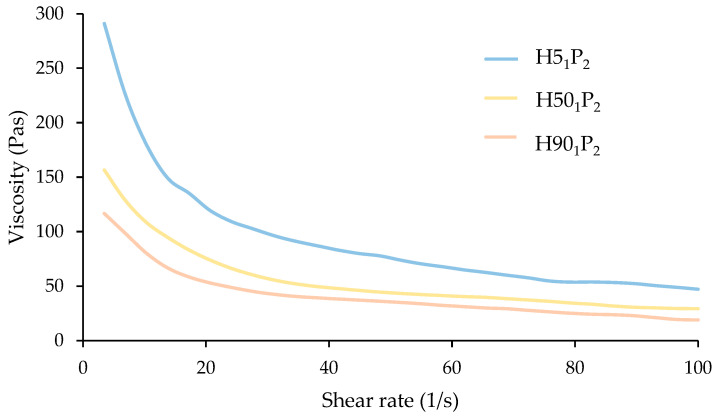
The relationship between apparent viscosity (Pas) and shear rate (1/s) of H5_1_P_2_, H50_1_P_2_, and H90_1_P_2_.

Polymer solution viscosity might affect the time required to dry an MN patch. Low-viscosity polymer solutions illustrate a fast evaporation rate [24]: low-viscosity solutions show a smooth surface unlike high-viscosity solutions, whose surface is more prone [25]. In this work, the solution viscosity depends on the loading content of PVP K90 in the formulation, as PVP K90 is a long-chain polymer having a complex intermolecular structure, obstructing the mobility of HPMC and drug molecules. Moreover, the high viscosity of the polymer solution also suppresses the filling of the small cavities in the PDMS mold with polymer solution [26]. In addition, the apparent viscosity of all polymer solutions decreased when the shear rate increased, as shown in Figure 4, so the centrifugation applied in this work facilitated the polymer solutions to be loaded into the MN molds [27].

#### 3.3.2. Morphological Structure of Microneedle Patch

**Figure 5:**
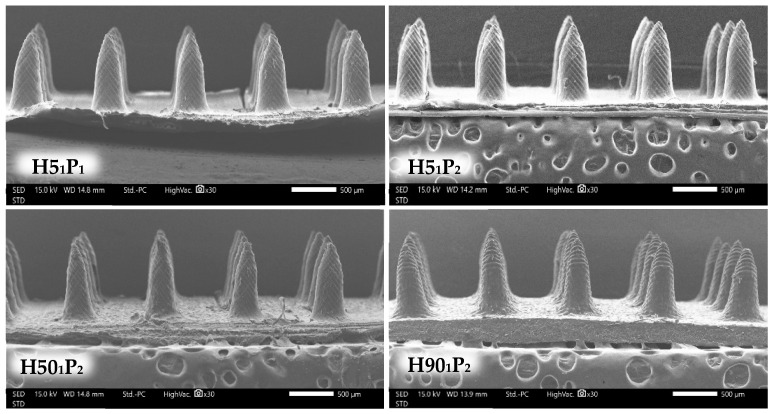
SEM images of MN structures of H5_1_P_1_, H5_1_P_2_, H50_1_P_2_, and H90_1_P_2_ at 30× magnification.

The MN structure was evaluated using SEM at 30× magnification in order to select the formulation with an acceptable physical appearance (Figure 5). The vital factor of dissolving MN is the ability to pierce through the skin, since they must be able to perforate the stratum corneum to deliver the drug in question. An improper physical appearance of the needles, such as broken and damaged needles, indicates poor mechanical strength, affecting the skin insertion ability of the MN [28]. Interestingly, the formulations that have only HPMC, which are H5, H50, and H90, showed unacceptable appearance, such as broken tips and unshaped needles, as shown in Table S1. The addition of PVP K90 elevates the hardness and toughness of the formulation [29], as indicated by the needles’ regular sharp shape. Although H5_2_P_1_, H50_1_P_1_, H50_2_P_1_, H90_1_P_1_, and H90_2_P_1_ comprised PVP K90, the shape of the needles was not as desired due to the inadequate amount of PVP K90 in the formulation [30]. Importantly, H5_1_P_1_ had a complete needle structure unlike H50_1_P_1_ and H90_1_P_1_, which had the same ratio of PVP K90 because H5_1_P_1_ had the highest amount of HPMC among these three formulations. Hence, the formulations that showed a complete MN structure were H5_1_P_1_, H5_1_P_2_, H50_1_P_2_, and H90_1_P_2_, as shown in Figure 5. These four formulations had no structural break or bend tips and also do not have holes around the needles. Moreover, the MN height and width of these four formulations were evaluated using ImageJ software version 18.0 for a comparison with MN mold, as shown in Table 2.

#### 3.3.3. Mechanical Property of Microneedle Patch

The mechanical property of MN patches was evaluated to ensure that the MN can pierce through the epidermis layer without a structural break. Mechanical strength was preliminarily evaluated to exclude the formulations that were fragile during the compression using the texture analyzer [28], and the given force was from 6.3 to 24.75 N. H5_1_P_1_ showed the highest % height change (11.53 ± 1.12%) due to the lowest amount of PVP K90 in the formulation. Literally, PVP K90 has great hardness and toughness; thus, the addition of PVP K90 raises the mechanical strength and reduces the fragility of the dissolving MN [5,30]. Although H5_1_P_2_, H50_1_P_2_, and H90_1_P_2_ had the same amount of PVP K90 in the formulation, the difference in the type and amount of HPMC affects the mechanical property. Firstly, different types of HPMC have different % bulky groups, which are the methoxy group and hydroxypropyl group, affecting the steric effect and resulting in a rise in mechanical strength, and the high molecular weight HPMC has higher % bulky groups than the low molecular weight HPMC. To be more precise, both the methoxy group and hydroxypropyl group make HPMC chains bulkier than the initial hydroxy groups, so they require higher the mechanical force to break the structure [31]. Secondly, an increasing amount of HPMC in the formulation improves the mechanical strength because the numerous HPMC chains can form a physical entanglement and intermolecular as well as intramolecular hydrogen bonds, resulting in a stronger network structure that requires high mechanical force to break the structure [32]. The % height changes in H5_1_P_2_, H50_1_P_2_, and H90_1_P_2_ were 6.29 ± 0.51, 6.13 ± 0.70 and 8.58 ± 0.86%, respectively (Table 3), and these three formulations exhibited great mechanical strength, since the % height change is less than 10% [33,34].

#### 3.3.4. Ex Vivo Skin Insertion Study of Microneedle Patch

**Figure 6:**
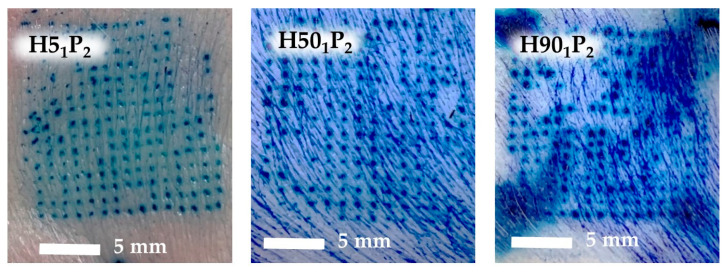
Skin insertion capability of H5_1_P_2_, H50_1_P_2_, and H90_1_P_2_.

Skin insertion was performed to ensure that the MN can pierce through the epidermis layer, since insertion ability is the important factor of the dissolving MN [28]. The factors affecting skin insertion were the sharpness of the needles, the type of polymers, and the characteristics of the skin tissues [24]. The blue dots appeared on the neonatal porcine skin, indicating a successful insertion of the MN through the skin, as shown in Figure 6, because the methylene blue is dry in the inserted holes [5]. It was found that the H5_1_P_2_ had the highest % blue dots of 81.89 ± 3.18%, which was followed by H50_1_P_2_ (77.51 ± 2.29%), H90_1_P_2_ (50.11 ± 1.94%), and H5_1_P_1_ (27.56 ± 0.87%), respectively (Table 3). Moreover, the % blue dots related to the mechanical property of the MN patch. Due to the low amount of PVP K90 in H5_1_P_1_ (20.00% *w*/*w*), unlike the other three formulations (26.67% *w*/*w*), H5_1_P_1_ had low % blue dots, because PVP K90 in the formulation impacted the strength and toughness of the needles. Thus, the addition of a higher amount of PVP K90 elevates the needle hardness, allowing the needles to pierce through the skin [29]. In particular, the MN patch having a low % height change exhibited high % blue dots.

### 3.4. Characterization of Lidocaine HCl-Incorporated MN

**Figure 7:**
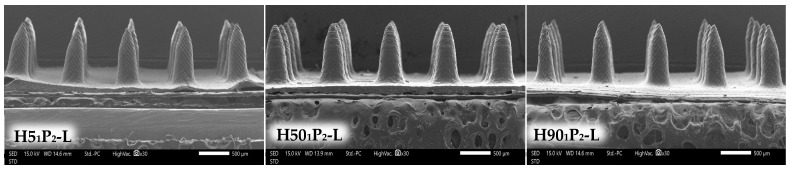
SEM images showing the needle physical morphology of H5_1_P_2_-L, H50_1_P_2_-L, and H90_1_P_2_-L at 30× magnification.

According to the physicochemical property results, the formulations with complete needles, low % height change, and high % blue dots were selected for the incorporation of lidocaine HCl (H5_1_P_2_, H50_1_P_2_, and H90_1_P_2_). On the other hand, H5_1_P_1_ was not selected for the lidocaine HCl incorporation due to its low % blue dots (27.56 ± 0.87%) and high % height change (11.53 ± 1.12%). The findings suggested that three new formulations (H5_1_P_2_-L, H50_1_P_2_-L, and H90_1_P_2_-L) showed complete sharp needles without a broken structure and holes nor bend tips, as shown in Figure 7, similar to the blank MN patch. It was worth noting that lidocaine HCl incorporation into the MN patch did not influence the needle’s physical appearance.

After lidocaine HCl-incorporated MN patches were pressed using the texture analyzer, the % height changes in H5_1_P_2_-L, H50_1_P_2_-L, and H90_1_P_2_-L were 5.96 ± 0.50, 5.68 ± 0.58, and 8.01 ± 0.63%, respectively (Table 4), and the results was acceptable since the % height change is less than 10% [33,34]. The % blue dots of those three formulations were 83.56 ± 1.94, 81.33 ± 2.04, and 80.44 ± 1.94%, respectively. The results of % height change and % blue dots were not significantly different from those of their blank MN patches, as shown in Table 3. Hence, the lidocaine HCl incorporation did not compromise the mechanical property and skin insertion ability of the MN patches.

### 3.5. Drug-Loading Capacity

The concentration of lidocaine HCl was selected according to the concentration available on the market (2–5% *w*/*w*). In this work, the 5% *w*/*w* lidocaine HCl concentration was selected for the studied formulations, and the added amount of lidocaine HCl was calculated from the weight of the polymer in the formulation. It was found that the % lidocaine HCl loading content in H5_1_P_2_-L, H50_1_P_2_-L, and H90_1_P_2_-L was 100.95 ± 9.21, 97.32 ± 5.71, and 102.66 ± 9.47%, respectively, as shown in Table 5. These three formulations show no significant difference in lidocaine HCl loading content.

### 3.6. Dissolving Study of MN

Dissolution is a vital parameter governing the MN dissolving performance. An ideal MN must dissolve when in contact with biological fluid and then release the drug or substance [35]. The change in the MN structure over time at 0, 5, 15, 30, and 60 min is shown in Table 6. The optical photographs showed that H90_1_P_2_-L and H50_1_P_2_-L completely dissolved after 15 and 60 min, respectively, while H5_1_P_2_-L virtually dissolved after 60 min. The dissolution time of the dissolving MN depended on the amount of methoxy group in HPMC. Specifically, HPMC containing high methoxy content showed a slow dissolution time, whereas low methoxy content in HPMC resulted in a fast dissolution time [36]. Here, HPMC 90SH had the lowest methoxy content of 19.0–24.0% *w*/*w*; thus, H90_1_P_2_-L exhibited the highest dissolution time within 15 min. However, HPMC E5 and HPMC E50 having the same amount of methoxy content (approximately 28.0–30.0% *w*/*w*) showed different dissolution times because the amount of HPMC in H5_1_P_2_-L is higher than that in H50_1_P_2_-L. Thus, the dissolution time of the H50_1_P_2_-L was accordingly faster than that of H5_1_P_2_-L.

### 3.7. Lidocaine HCl Release

**Figure 8:**
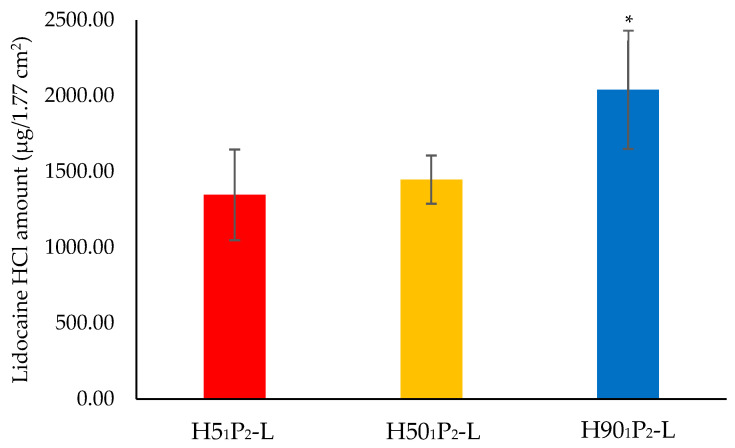
Deposited amount of lidocaine HCl in porcine skin after 1 h. The results are illustrated as mean ± S.D. at a significant level of * (*p* < 0.05).

Lidocaine HCl release was performed to measure whether the amount of lidocaine HCl in the piercing area remained sufficient, inducing an anesthetic effect. The results illustrated that the deposit amounts of lidocaine HCl in skin from H5_1_P_2_-L, H50_1_P_2_-L, and H90_1_P_2_-L were 1346.92, 1447.36 and 2040.79 μg/1.77 cm^2^, as shown in Figure 8. Moreover, the results showed that H90_1_P_2_-L had the highest amount of released lidocaine HCl, which was a significant difference from H5_1_P_2_-L and H50_1_P_2_-L. At 60 min, the results had related to dissolving ability, since H90_1_P_2_-L showed the fastest dissolving ability. Thus, H90_1_P_2_-L should release the highest amount of lidocaine HCl, while the needles of H5_1_P_2_-L and H50_1_P_2_-L did not completely dissolve, so the released amount of lidocaine HCl was lower than for H90_1_P_2_-L. Interestingly, all three formulations provided an adequate released amount of lidocaine HCl to reach an anesthetic effect (approximately 135 μg/cm^2^ or 239 μg/1.77 cm^2^) [20]. Moreover, the released amount of lidocaine HCl will not cause the side effects since the toxic dose of lidocaine HCl is when the dose exceeds 4.5 mg/kg [37].

### 3.8. Fourier Transform Infrared Spectroscopy

**Figure 9:**
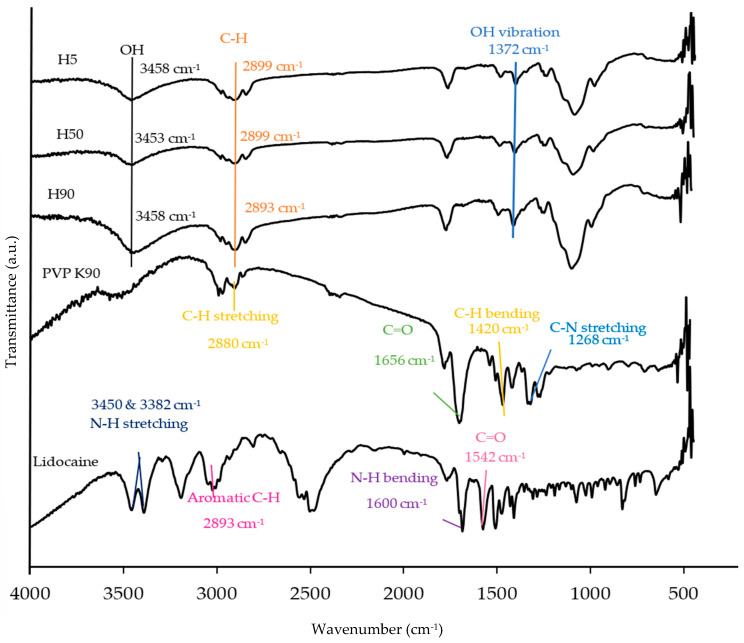
FTIR spectra of H5, H50, H90, PVP K90, and lidocaine HCl in transmittance mode from 400 to 4000 cm^−1^.

**Figure 10:**
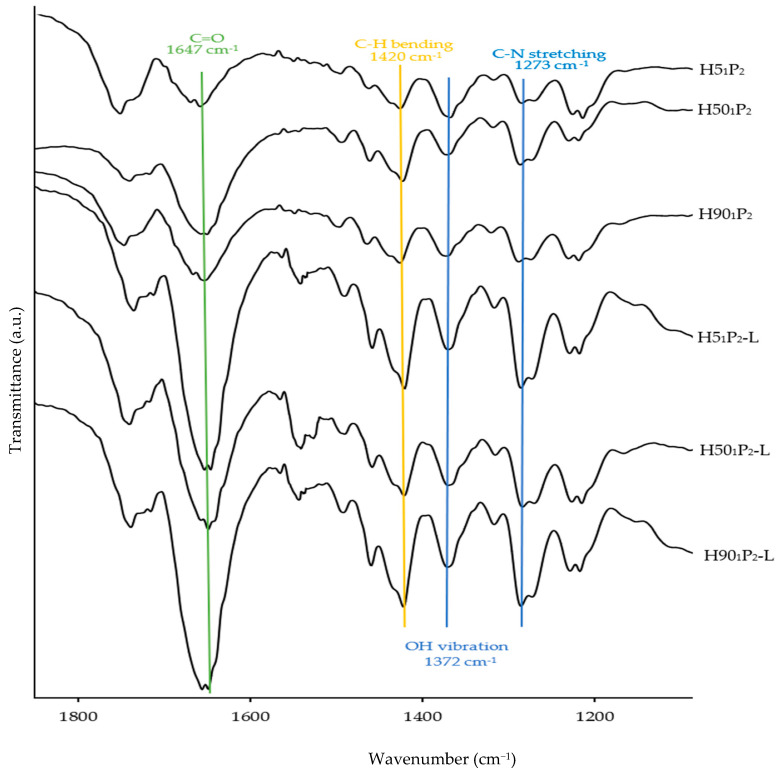
FTIR spectra of H5_1_P_2_, H50_1_P_2_, H90_1_P_2_, H5_1_P_2_-L, H50_1_P_2_-L, and H90_1_P_2_-L in transmittance mode from 1200 to 1800 cm^−1^.

Figure 9 illustrates the important functional groups of H5, H50, H90, PVP K90, and lidocaine HCl determined by the FTIR technique. The FTIR full spectra of H5_1_P_2_, H50_1_P_2_, H90_1_P_2_, H5_1_P_2_-L, H50_1_P_2_-L, and H90_1_P_2_-L in a transmittance mode from 400 to 4000 cm^−1^ are shown in Figure S1. The chemical shift or peak intensity change in FTIR spectra reflected the possible interactions between the polymers [9]. The important peaks of HPMC with different grades (H5, H50 and H90) were assigned to -OH stretching, C-H stretching and -OH vibration at ~3458, 2899, and 1372 cm^−1^, respectively [38,39,40,41]. There were four major peaks in the PVP K90 spectrum: C-H stretching vibration, C=O stretching, C-H bending, and C-N stretching at 2880, 1656, 1420 and 1268 cm^−1^, respectively [42,43]. The lidocaine HCl spectrum showed N-H stretching of amine salt at 3450 and 3382 cm^−1^, aromatic C-H stretching at 2893 cm^−1^, N-H bending at 1600 cm^−1^, and C=O stretching at 1542 cm^−1^ as the important peaks [44,45]. In Figure 10, the blending of PVP K90 with various grades of HPMC in ethanol and DI water mixed in a ratio of 8:2 did not cause a significant change in the FTIR spectra of H5_1_P_2_, H50_1_P_2_, and H90_1_P_2_ as compared to those of the parent polymers (Figure 9). The all-important peaks of the blends remained at the same wavenumbers as the two polymers: -OH vibration at 1372 cm^−1^ from HPMC, including C=O stretching at 1647 cm^−1^, C-H bending at 1420 cm^−1^ and C-N stretching at 1273 cm^−1^ from PVP K90. In the presence of lidocaine HCl in the HPMC/PVP K90 blends, N-H bending (1600 cm^−1^) from lidocaine HCl cannot be evaluated due to the interfering peaks between N-H bending (1600 cm^−1^) from lidocaine HCl and C=O bending (1654 cm^−1^) from PVP K90. Although N-H bending from lidocaine HCl at 1600 cm^−1^ cannot be evaluated, the corresponding peak of N-H bending from lidocaine HCl and C=O stretching from PVP K90 can be used to identify the present of lidocaine HCl in the formulation, since the corresponding areas of these two peaks indicated a high-intensity peak at 1647 cm^−1^. As a result, lidocaine HCl was successfully loaded into the formulations and presented in the dissolving MN. For the H5_1_P_2_-L, H50_1_P_2_-L, and H90_1_P_2_-L spectra, there were no significant chemical peak shifts, indicating no chemical or physical interactions between the two polymers and lidocaine HCl [9]. Therefore, the lidocaine HCl delivery from the MN patches was not hindered by the HPMC and PVP K90.

## 4. Conclusions

Three-dimensional printing was demonstrated as an alternative method to construct an MN patch instead of micro-electromechanical systems. MN patch masters were initially 3D-printed from acrylic-based resin, which was followed by the fabrication of reverse PDMS MN molds, which were used as templates for constructing HPMC/PVP K90 MN patches. Dissolving MN comprising HPMC alone showed an irregular needle shape. Enhancement of the MN mechanical strength was achieved by blending PVP K90 with the HPMC, resulting in a low % height change and high % blue dots. The HPMC/PVP K90 dissolving MN had a complete structure with sharp tips, which was capable of exhibiting high % lidocaine HCl loading content and providing a fast-dissolving property. Importantly, the HPMC/PVP K90 dissolving MN that incorporated lidocaine HCl provided a sufficient amount of released lidocaine HCl in the skin. In addition, FTIR analysis results indicated no chemical interactions between the two polymers and lidocaine HCl. Consequently, the drug delivery was not restricted by the compositions within the MN patches. Since HPMC and PVP K90 are safe to use in humans, the HPMC/PVP K90 dissolving MN patches were promising devices for lidocaine HCl delivery through skin with safety and efficacy. Future investigation of this work would be employing an animal study to determine the pharmacodynamics of the developed HPMC/PVP K90 dissolving MN.

## Figures and Tables

**Table 1:** The microneedle compositions with different formulations (30 g).

Sample Code	HPMC			PVP K90 40% (g)	EtOH:H_2_O (8:2) q.s. to (g)
	E5 20% (g)	E50 7% (g)	E90 1.5% (g)		
H5	6.00	-	-	-	30.00
H5_1_P_1_	3.00	-	-	6.00	
H5_1_P_2_	2.00	-	-	8.00	
H5_2_P_1_	4.00	-	-	4.00	
H50	-	2.10	-	-	
H50_1_P_1_	-	1.05	-	6.00	
H50_1_P_2_	-	0.70	-	8.00	
H50_2_P_1_	-	1.40	-	4.00	
H90	-	-	0.45	-	
H90_1_P_1_	-	-	0.23	6.00	
H90_1_P_2_	-	-	0.15	8.00	
H90_2_P_1_	-	-	0.30	4.00	

**Table 2:** Height change (%) and width change (%) of H5_1_P_1_, H5_1_P_2_, H50_1_P_2_, and H90_1_P_2_ compared to the dimension of the MN mold.

Sample Code	Height (μm)	% Height Change	Width (μm)	% Width Change
H5_1_P_1_	866.51 ± 7.44 ^a^	3.19 ± 2.52	418.84 ± 12.26 ^a^	7.24 ± 3.25
H5_1_P_2_	876.95 ± 9.68 ^a,b^	2.02 ± 2.88	438.48 ± 11.42 ^b^	2.98 ± 4.52
H50_1_P_2_	881.49 ± 8.26 ^b^	1.51 ± 3.02	431.82 ± 13.11 ^a,b^	4.42 ± 5.51
H90_1_P_2_	878.06 ± 8.55 ^b^	1.90 ± 2.79	437.52 ± 17.42 ^b^	3.19 ± 2.35

For each test, the average values with the same letter show no statistical difference (*p* > 0.05).

**Table 3:** Height change (%) after compression by the texture analyzer and blue dots (%) of H5_1_P_1_, H5_1_P_2_, H50_1_P_2_, and H90_1_P_2_.

Sample Code	H_1_ (μm)	H_2_ (μm)	% Height Change	% Blue Dots
H5_1_P_1_	866.93 ± 8.95	766.96 ± 9.03	11.53 ± 1.12 ^a^	27.56 ± 0.87 ^a^
H5_1_P_2_	875.56 ± 9.91	820.50 ± 10.95	6.29 ± 0.51 ^b^	81.89 ± 3.18 ^b^
H50_1_P_2_	878.46 ± 7.14	824.58 ± 4.26	6.13 ± 0.70 ^b^	78.51 ± 2.29 ^b^
H90_1_P_2_	875.66 ± 6.30	800.54 ± 10.50	8.58 ± 0.86 ^c^	77.11 ± 1.94 ^b^

For each test, the average values with the same letter show no significant difference (*p* > 0.05).

**Table 4:** Height change (%) after compression by the texture analyzer and blue dots (%) of H5_1_P_2_-L, H50_1_P_2_-L, and H90_1_P_2_-L.

Sample Code	H_1_ (μm)	H_2_ (μm)	% Height Change	% Blue Dots
H5_1_P_2_-L	867.45 ± 7.08	815.71 ± 7.00	5.96 ± 0.50 ^a^	83.56 ± 1.94 ^a^
H50_1_P_2_-L	872.85 ± 7.82	823.30 ± 8.83	5.68 ± 0.58 ^a^	81.33 ± 2.04 ^a^
H90_1_P_2_-L	874.25 ± 9.16	804.20 ± 6.06	8.01 ± 0.63 ^b^	80.44 ± 1.94 ^a^

For each test, the average values with the similar letter show no statistical difference (*p* > 0.05).

**Table 5:** Lidocaine HCl loading content (%) in H5_1_P_2_-L, H50_1_P_2_-L, and H90_1_P_2_-L.

Sample	% Lidocaine HCl Loading Content
H5_1_P_2_	100.95 ± 9.21 ^a^
H50_1_P_2_	97.32 ± 5.71 ^a^
H90_1_P_2_	102.66 ± 9.47 ^a^

For each test, the average values with similar letters show no statistical difference (*p* > 0.05).

**Table 6:** Dissolution images of H5_1_P_2_-L, H50_1_P_2_-L, and H90_1_P_2_-L at the dissolution times of 0, 5, 15, 30 and 60 min using neonatal porcine skin saturated with PBS pH 7.4.

Sample	Time (min)				
	0	5	15	30	60
H51P2-L	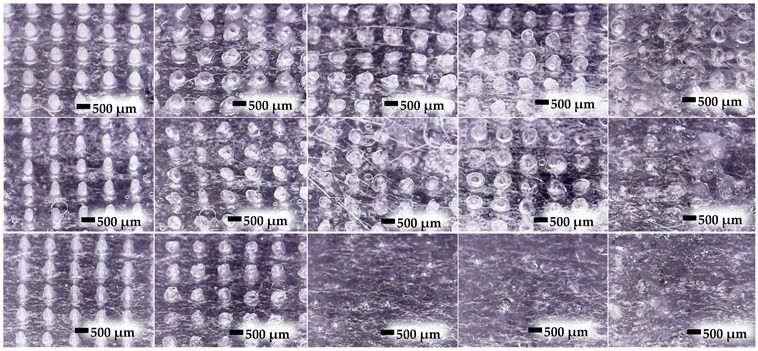				
H501P2-L					
H901P2-L					

## Data Availability

The authors confirm that the data are contained within the article and supplementary materials.

## Supplementary Materials

The following supporting information can be downloaded at https://www.mdpi.com/article/10.3390/polym16040452/s1, Table S1: MN structures of H5, H5_1_P_1_, H5_1_P_2_, H5_2_P_1_, H50, H50_1_P_1_, H50_1_P_2_, H50_2_P_1_, H90, H90_1_P_1_, H90_1_P_2_, H90_2_P_1_ using SEM at 30× magnification, Figure S1: FTIR spectra of H5, H50, H90, PVP K90, lidocaine HCl, H5_1_P_2_, H50_1_P_2_, H90_1_P_2_, H5_1_P_2_-L, H50_1_P_2_-L, and H90_1_P_2_-L in a transmittance mode from 400–4000 cm^−1^.
