# Supplementary material for: HPMC/PVP K90 Dissolving Microneedles Fabricated from 3D-Printed Master Molds: Impact on Microneedle Morphology, Mechanical Strength, and Topical Dissolving Property
Source: Polymers (Basel). 2024 Feb 6;16(4):452. doi: 10.3390/polym16040452 (PMC10891514; doi:10.3390/polym16040452)
Supplement: Supplementary file 1 [file polymers-16-00452-s001.zip › polymers-2823614-supplementary.pdf]

polymers

MDPI

Supplementary Materials

HPMC/PVP K90 Dissolving Microneedles Fabricated from 3D-Printed Master Molds: Impact on Microneedle Morphology, Mechanical Strength, and Topical Dissolving Property

Baramee Chanabodeechalermrung [1], Tanpong Chaiwarit [1], Siripat Chaichit [1], Suruk Udomsom [2,3,4], Phornsawat Baipaywad [2,3], Patnarin Worajittiphon [5,6] and Pensak Jantrawut [1,6,7,*]

1 Department of Pharmaceutical Sciences, Faculty of Pharmacy, Chiang Mai University, Chiang Mai 50200, Thailand; barameechana@gmail.com (B.C.); tanpong_c@cmu.ac.th (T.C.); siripat.chaichit@cmu.ac.th (S.C.)
2 Biomedical Engineering and Innovation Research Center, Chiang Mai University, Chiang Mai 50200, Thailand; suruk_u@cmu.ac.th (S.U.); phornsawat.b@cmu.ac.th (P.B.)
3 Biomedical Engineering Institute (BMEI), Chiang Mai University, Chiang Mai 50200, Thailand
4 Office of Research Administration, Chiang Mai University, Chiang Mai 50200, Thailand
5 Department of Chemistry, Faculty of Science, Chiang Mai University, Chiang Mai 50200, Thailand; patnarin.w@cmu.ac.th
6 Center of Excellence in Materials Science and Technology, Chiang Mai University, Chiang Mai 50200, Thailand
7 Center of Excellence in Agro Bio-Circular-Green Industry (Agro BCG), Agro-Industry, Chiang Mai University, Chiang Mai 50100, Thailand
* Correspondence: pensak.amuamu@gmail.com or pensak.j@cmu.ac.th; Tel.: +66-989569459

Table S1. MN structures of H5, $H5_1P_1$, $H5_1P_2$, $H5_2P_1$, H50, $H50_1P_1$, $H50_1P_2$, $H50_2P_1$, H90, $H90_1P_1$, $H90_1P_2$, $H90_2P_1$ using SEM at 30× magnification.

Sample Code	SEM (X 30)	Sample Code	SEM (X 30)
H5	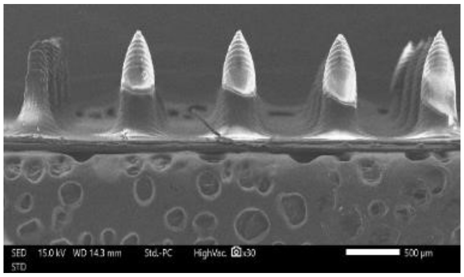	$H50_1P_2$	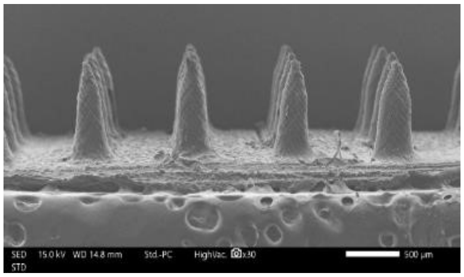
$H5_1P_1$	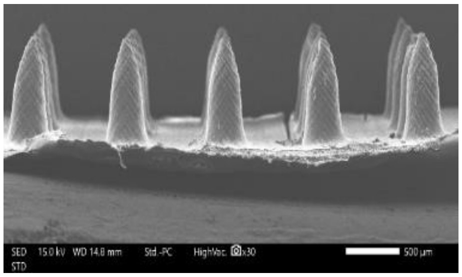	$H50_2P_1$	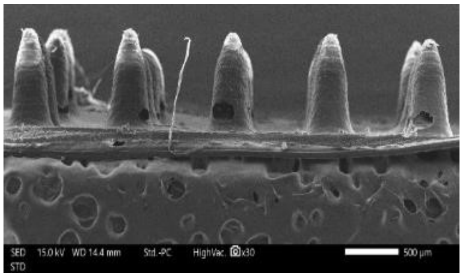

Table S1. MN structures of H5, $H5_1P_1$, $H5_1P_2$, $H5_2P_1$, H50, $H50_1P_1$, $H50_1P_2$, $H50_2P_1$, H90, $H90_1P_1$, $H90_1P_2$, $H90_2P_1$ using SEM at 30× magnification (cont.).

Sample Code	SEM (X 30)	Sample Code	SEM (X 30)
$H5_1P_2$	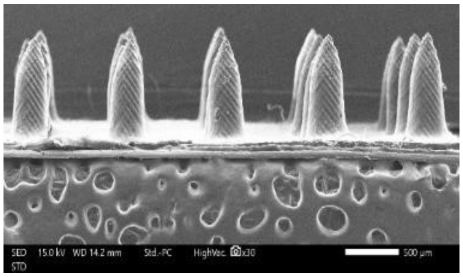	H90	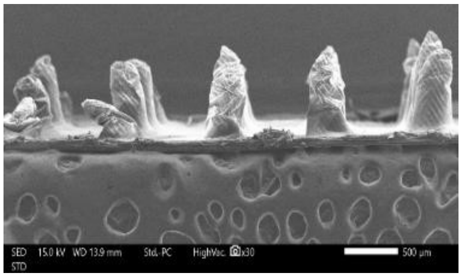
$H5_2P_1$	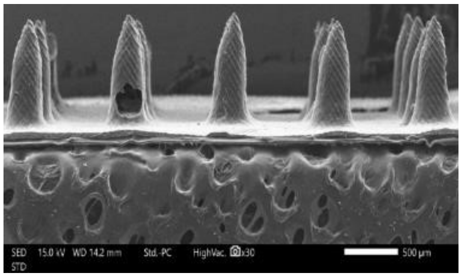	$H90_1P_1$	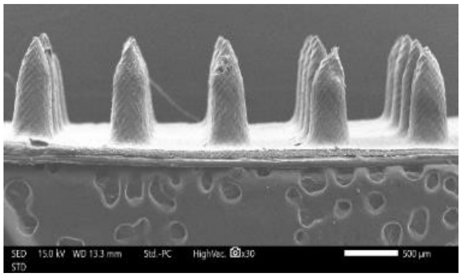
H50	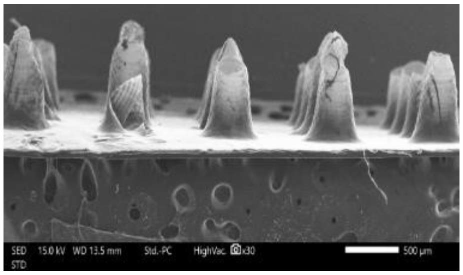	$H90_1P_2$	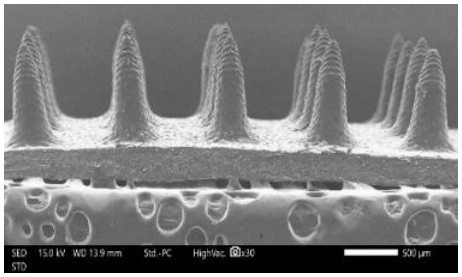
$H50_1P_1$	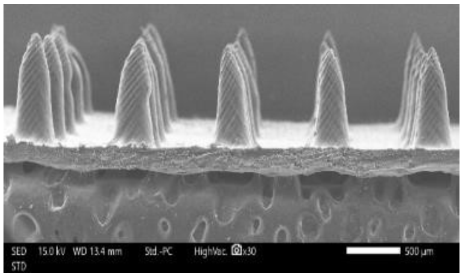	$H90_2P_1$	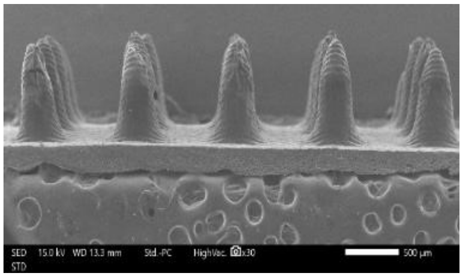

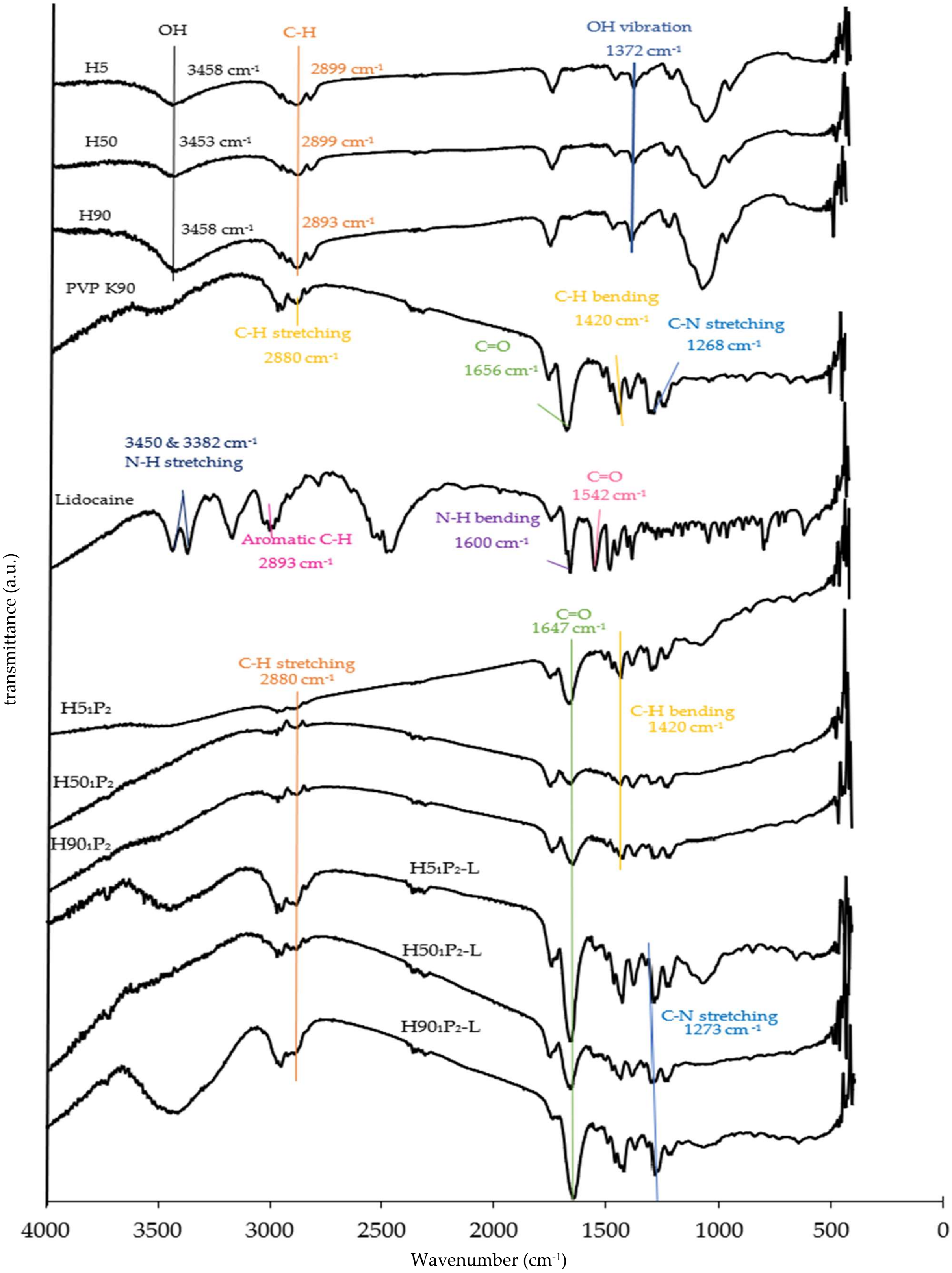

Figure S1. FTIR spectra of H5, H50, H90, PVP K90, lidocaine HCl, $H5_1P_2$, $H50_1P_2$, $H90_1P_2$, $H5_1P_2$-L, $H50_1P_2$-L, and $H90_1P_2$-L in a transmittance mode from 400-4000 cm^{-1}.
